# Chipless-RFID: A Review and Recent Developments

**DOI:** 10.3390/s19153385

**Published:** 2019-08-01

**Authors:** Cristian Herrojo, Ferran Paredes, Javier Mata-Contreras, Ferran Martín

**Affiliations:** 1 CIMITEC, Departament d’Enginyeria Electrònica, Universitat Autònoma de Barcelona, 08193 Bellaterra, Barcelona, Spain; 2 Departamento de Ingeniería de Comunicaciones, Escuela Técnica Superior de Ingeniería de Telecomunicación, Universidad de Málaga, 29071 Málaga, Spain

**Keywords:** chipless-RFID, coding techniques, frequency domain, time domain, hybrid chipless-RFID tags

## Abstract

In this paper, a review of the state-of-the-art chipless radiofrequency identification (RFID) technology is carried out. This recent technology may provide low cost tags as long as these tags are not equipped with application specific integrated circuits (ASICs). Nevertheless, chipless-RFID presents a series of technological challenges that have been addressed by different research groups in the last decade. One of these challenges is to increase the data storage capacity of tags, in order to be competitive with optical barcodes, or even with chip-based RFID tags. Thus, the main aim of this paper is to properly clarify the advantages and disadvantages of chipless-RFID technology. Moreover, since the coding information is an important aspect in such technology, the different coding techniques, as well as the main figures of merit used to compare different chipless-RFID tags, will be analyzed.

## 1. Introduction

Radiofrequency identification (RFID) is a wireless technology for identification and tracking of objects, animals, persons, etc. which has moderately penetrated the market, as compared to optical identification systems (based on barcodes or QR-codes) [1,2,3,4,5,6,7]. Despite the fact that RFID offers significant advantages over optical systems, such as larger reading distances without the need of line-of-sight, high data storage capacity, and the possibility to read multiple tags simultaneously, the cost of traditional RFID tags (i.e., tags equipped with a silicon integrated circuit or chip), is excessively high for many applications involving low-cost items. This applies even to passive tags, fed by the interrogation signal, where batteries are not present. Thus, the typical cost of passive UHF-RFID tags is situated in the vicinity of several eurocents, much beyond the cost of optical barcodes or QR codes. Since the cost of chipped RFID tags is mainly dictated by the presence of the chip, the implementation of planar encoders, able to replace the chip in the tag, has been an object of intensive research in the last years, giving rise to the so-called chipless-RFID technology [8,9,10,11,12,13]. Thus, in chipless-RFID tags, the tag is equipped with a planar encoder (typically a printed pattern containing the ID code) and sometimes with an antenna for communication with the reader. By using low-cost conductive inks, the price of the tag can be substantially reduced. However, with chipless-RFID it is not possible to be as competitive as with chipped-RFID in terms of system performance (read range, data capacity, etc.). Indeed, the main research activity relative to chipless-RFID technology in the last years has been oriented towards increasing the data storage capacity and density of the tags. In this review paper, the main strategies for the implementation of chipless-RFID systems are discussed, and the latest or more relevant implementations are highlighted. Such approaches include (i) time domain systems, (ii) frequency domain systems, and (iii) hybrid systems.

## 2. Chipless-RFID Tags Based on Time Domain

### 2.1. Time Domain Reflectometry Based Tags (TDR)

**Figure 1:**
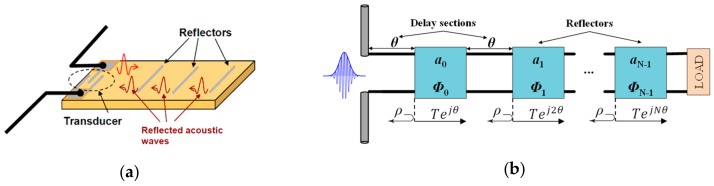
Working principle of a chipless radiofrequency identification (RFID) system based on (**a**) surface acoustic wave (SAW) technology and (**b**) delay lines. In both cases, the functionality is known as time domain reflectometry (TDR).

The former chipless-RFID tags were inspired by the operation of RFID tags which encode information in the time domain. Such tags were implemented on surface acoustic wave (SAW) technology [14,15,16,17,18,19] and basically consist of an electro-acoustic transducer connected to an antenna. The working principle is shown in Figure 1a. An electromagnetic pulse is sent by the reader which is received by the antenna and transformed into acoustic energy by the transducer. Then, the acoustic wave propagates through the substrate, reflecting part of it, in those positions where there is a reflector. Such reflections are converted back into electromagnetic energy and transmitted to the reader. This working principle is generally designated as time domain reflectometry (TDR), and the ID code is contained in the echoes generated by the reflectors. It is important to mention that chipless-RFID tags based on SAW technology provide high information capacity (up to 256 bits) [20]. Nevertheless, such tags are not competitive in terms of cost, since tag price may vary around 10–20 euro cents [2].

One solution, with a very similar operating principle (see Figure 1b), consists of implementing chipless-RFID tags composed of a delay line (transmission line), on conventional microwave substrates, which contain discontinuities (as reflectors) or complex impedances at certain positions. Basically, a pulse is sent by the reader which propagates through the delay line. Such a pulse is reflected at those points where there is a discontinuity. In this way, the identification code is constituted by the reflections or echoes produced in the tag. It is important to mention that the speed of sound is 340 m/s, one million times smaller than the speed of light (*c*). This aspect implies either implementing transmission lines by means of large lengths to produce measurable delays or very narrow pulses in order to avoid overlapping between reflected pulses. Such requirements are difficult to achieve in practice; therefore, the bit encoding capability is generally limited in chipless-RFID tags based on time domain. Nevertheless, the most common codification techniques used in time domain chipless-RFID systems are shown below.

#### 2.1.1. On-Off Keying (OOK)

**Figure 2:**
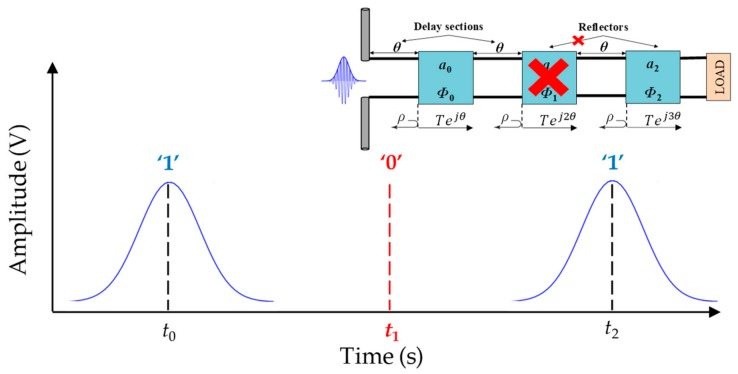
Working principle of on-off keying encoding.

On-off keying (OOK) encoding is considered to be the simplest encoding method in time domain-based chipless-RFID systems. Basically, the logic states ‘0’ and ‘1’ are determined by the absence or presence, respectively, of a reflection of the interrogation signal in a specific time interval, as illustrated in Figure 2.

**Figure 3:**
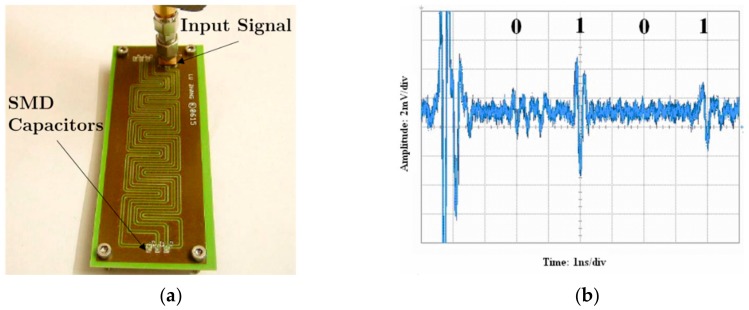
(**a**) Photograph of the chipless-RFID tag proposed by Zhang et al. (code ‘0101’); (**b**) measured code ‘0101’. Figure extracted from [21].

The OOK encoding method was reported for the first time by Zhang et al. [21] in 2006. The tag is composed of a meandered coplanar waveguide transmission line (CPW) with surface mount device (SMD) capacitors inserted in parallel at predefined positions (see Figure 3a). Such capacitors or discontinuities act as reflectors. A pulse of short duration, specifically 2 ns, which propagates through the transmission line CPW, is sent by the reader. The presence of a capacitive discontinuity at certain positions produces a mismatching that results in a reflected pulse which is transmitted back to the reader. The presence of such reflection is interpreted as the logic state ‘1’, whereas its absence is associated to logic state ‘0’. An example of a measured 4-bit tag is shown in Figure 3b.

**Figure 4:**
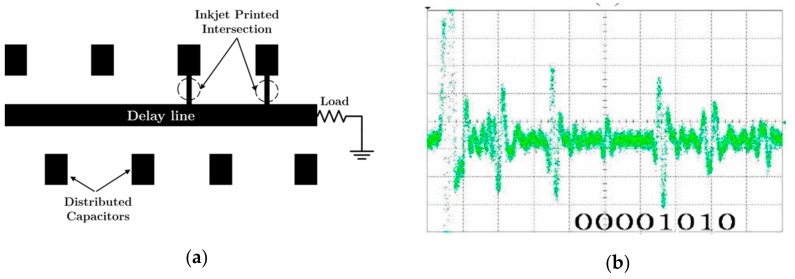
(**a**) Layout of the chipless-RFID tag proposed by Zheng et al. programmed with the code ‘00001010’ and (**b**) measurement of the code ‘00001010’. Figure extracted from [22].

Zheng et al. [22] and Botao et al. [23], in 2008 and 2010, respectively, replaced the SMD capacitors by patches, in order to obtain a fully planar structure. In this case, such patches were printed during tag fabrication and disconnected from the microstrip transmission line, as can be seen in Figure 4a. Consequently, the identification code of the tag is programmed by means of the connection of certain patches to the delay line. In order to reduce the size of the tag by approximately a factor of four, using the same substrate as in [21], the pulse duration of the interrogation signal was reduced to 0.4 *n*s. An example of a measured 8-bit tag is shown in Figure 4b.

**Figure 5:**
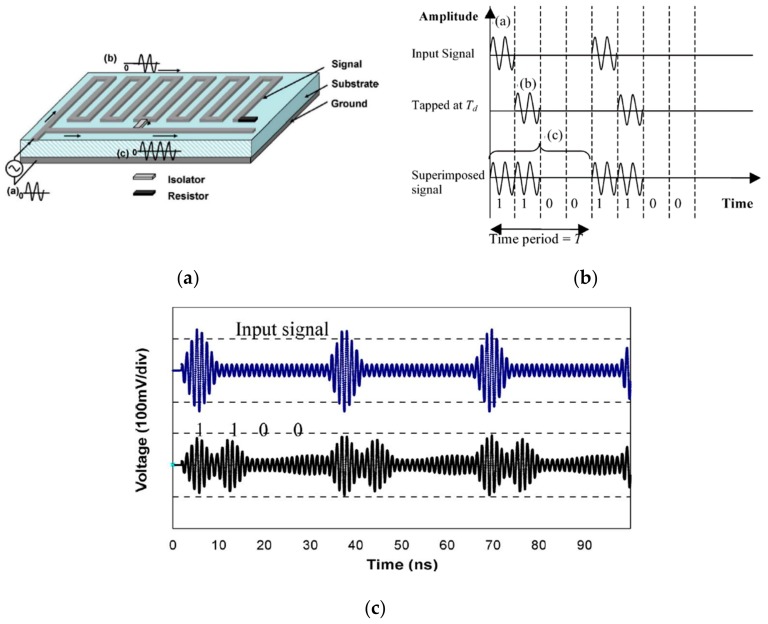
(**a**) Sketch of the delay line proposed by Chamarti et al.; (**b**) binary code generation by the superposition of delayed lines; (**c**) simulated input and output signals. Figure extracted from [24].

In [24,25] a different strategy to implement chipless-RFID tags based on time domain with OOK coding was reported. The main novelty is the fact that the delay line is used in transmission, rather than in reflection. As shown in Figure 5a, the input signal is divided into two branches, a short and straight line (without delay), and the other one a longer and meandered line (with delay). The two branches are connected by a circulator (which acts as an isolator) to deflect the second pulse at the appropriate time. Thus, the temporal position of the pulse is modified with the position of the isolator, and consequently, the encoding of the tag. In order to clarify the encoding strategy, Figure 5b,c show the principle of code generation, based on the superposition of delayed signals, and a simulated code example, respectively.

**Figure 6:**
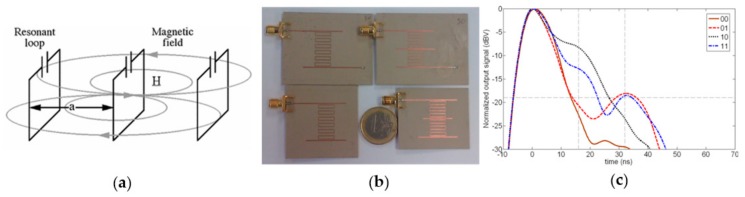
(**a**) Magneto inductive waveguide which consists of a set of capacitively loaded loops, magnetically coupled to each other. Figure extracted from [30]; (**b**) photograph of the fabricated set of 2-bit chipless RFID tags proposed by Herraiz et al. and (**c**) measured response. Figure extracted from [26].

On the other hand, in 2012, a novel strategy based on delay lines able to support magneto-inductive waves (MIW), was proposed by Herraiz et al. [26,27,28]. As depicted in Figure 6a, MIWs are slow waves that propagate through a chain of magnetically coupled resonators [26,29]. Such slow waves exhibit a group velocity (*v_g_*) around *c*/100 (where *c* is the speed of light in vacuum), hence maximizing the group delay (*τ_g_*). Thus, for the same delay, shorter delay lines can be achieved (as compared to conventional lines). For the implementation of the delay lines, split ring resonators (SRRs) with rectangular topology were used to maximize the coupling. A microstrip transmission line coupled inductively to the first resonator of the chain of SRRs, acts as a transducer between the microstrip mode and the MIW mode of the delay line. Finally, the additional microstrip transmission lines are used as reflectors, and coupled to specific SRRs of the chain in order to generate a specific identification code. Thereby, the position of the reflectors is different for each tag (see Figure 6b), giving rise to different temporal responses, as appreciated in Figure 6c. The main limitation of this strategy is the fact that the high losses of the MIW delay lines prevent the implementation of tags with high information capacity.

#### 2.1.2. Pulse Position Modulation

**Figure 7:**
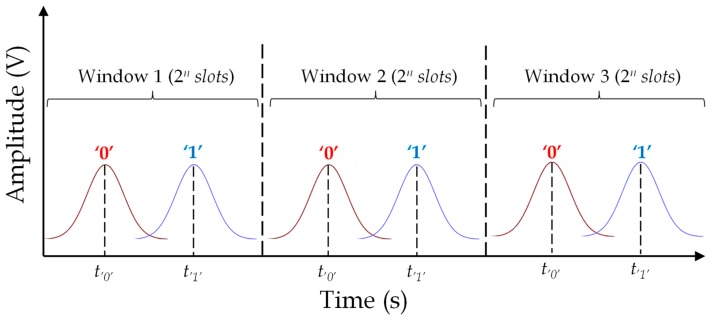
Working principle of pulse position modulation encoding.

Pulse position modulation (PPM) encoding consists of the variation of the position of a pulse within a certain temporal window. Specifically, PPM encodes *n* bits with a single pulse, which occupies one of the 2*^n^* slots of a temporal window (see Figure 7). The advantage of this encoding, with respect to OOK, is that it requires a smaller number of reflectors. Nevertheless, a longer time span is necessary in order to achieve the same information capacity.

**Figure 8:**
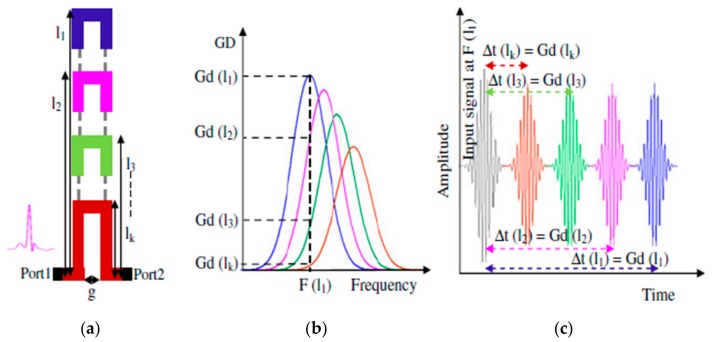
Principle of encoding by means of group delay: (**a**) structure of the chipless-RFID tag; (**b**) group delay vs. frequency for different lengths of C-sections, and (**c**) the corresponding time domain response. Figure extracted from [34].

Most of the works reported in the literature that use PPM encoding use dispersive structures which exhibit a frequency-dependent group velocity. Thus, a broadband signal propagating through such structures experiences a different group delay for each spectral component [31,32]. This structure can be implemented by means of C-section all-pass networks [33], which are composed of two coupled lines (see Figure 8a), whose electrical length is *λ_g_*/4 at the frequency where the group delay is maximum. Therefore, the frequency position of such maximum depends on the length of the structure, as depicted in Figure 8b. Consequently, in the time domain, for the corresponding frequency, a pulse delay equal to the group delay was implemented.

**Figure 9:**
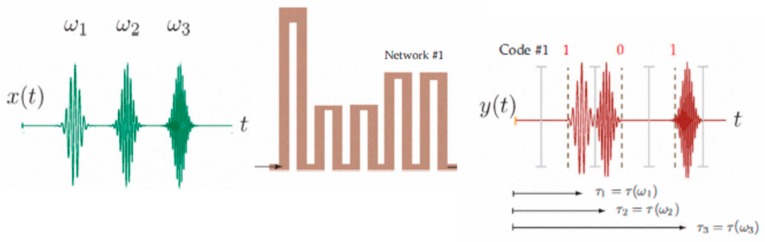
Chipless-RFID tag proposed by Gupta et al. fed with three Gaussian pulses with frequency ω_1_, ω_2_, and ω_3_ and an example of PPM encoding. Figure extracted from [35].

In 2010, a 3-bit tag based on cascaded C-section dispersive structures was implemented for the first time by Gupta et al. [35] (see Figure 9). Such a tag was implemented in order to provide maximums in the group delay at 3, 4, and 5 GHz. In this case, each pulse can provide two different positions within a temporal window, depending on its encoding (‘0’ or ‘1’). Other examples based on this concept can be found in [34,35,36,37].

#### 2.1.3. Phase Modulation

**Figure 10:**
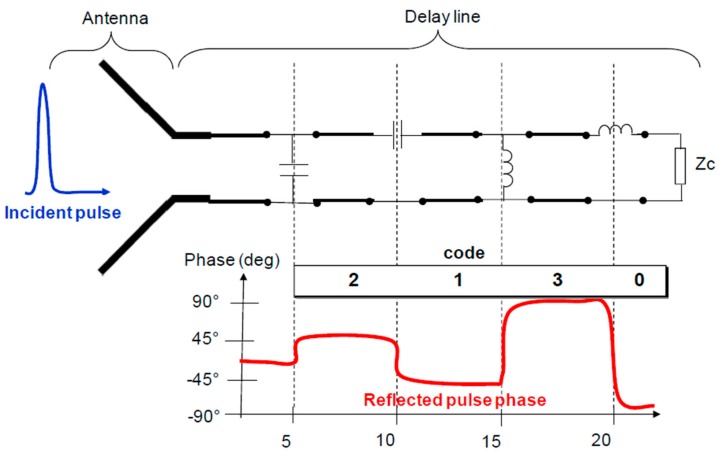
Temporal encoding based on the phase modulation of the input pulse to the tag. Figure extracted from [3].

In order to increase the information density of the tag, a different coding method based on the phase modulation of the input pulse to the tag was proposed by Mandel et al. [38,39]. Moreover, such tags include the use of composite right/left handed (CRLH) artificial delay lines, also used in [13,40]. The use of these lines is justified by their lower group velocity, as compared to conventional delay lines. This aspect allows for the implementation of shorter delay lines for a specific delay. In this case, complex loads of different values are used for the purpose of modifying the phase of the reflected pulses, as can be seen in Figure 10. As a result, with four different elements it is possible to achieve four different phase values (2 bits for each reflector). The number of codes that can be generated is, therefore, 4*^k^* (where *k* is the number of reflectors of the delay line).

### 2.2. Time Division Multiplexing Based Tags

**Figure 11:**
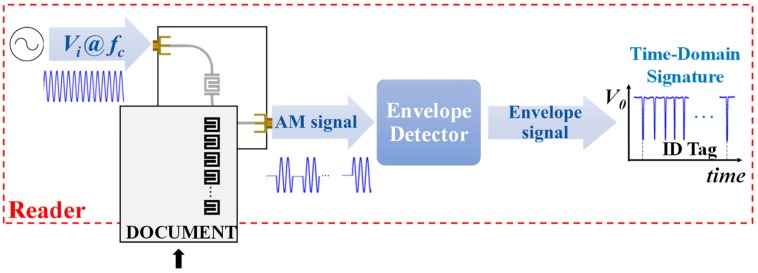
Sketch of the working principle of time domain chipless-RFID systems based on near-field coupling and sequential bit reading.

**Figure 12:**
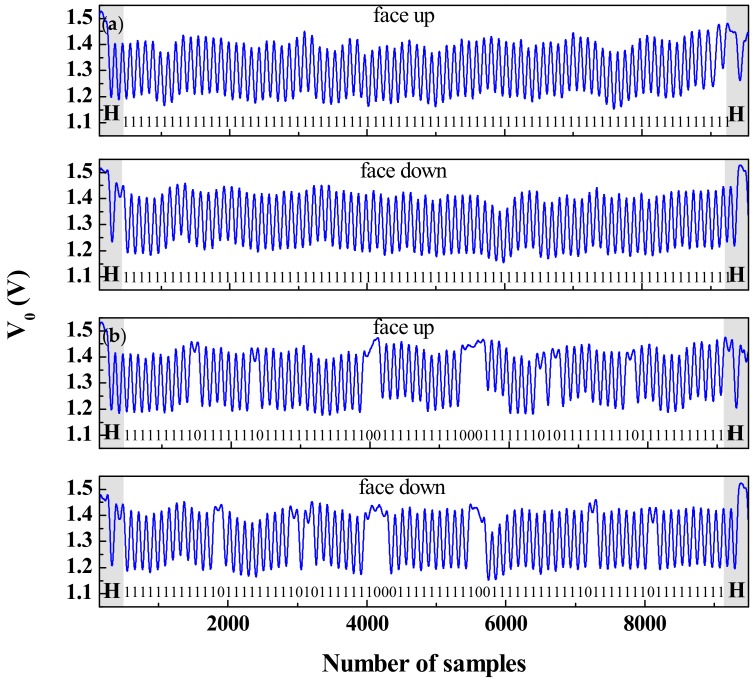
(**a**) Measured envelope of the inkjet-printed 80-bit tag with all bits set to the logic state ‘1’ and (**b**) 80-bit programmed tag with the indicated code. Figure extracted from [48].

Time division multiplexing is a novel and unconventional strategy to implement chipless-RFID systems based on time domain, which is of particular interest in secure paper applications. Nevertheless, the working principle is completely different than those of the aforementioned chipless-RFID systems. Such a strategy was reported for the first time by Herrojo et al. in 2017 [41]. With this approach, the number of achievable bits is only limited by the area occupied by the tags and the ID code is contained in the amplitude modulated signal generated by the tags. Such tags typically consist of a chain of either resonant elements [42,43,44,45,46,47,48,49] or metallic strips [50,51,52], etched or printed at predefined and equidistant positions on a dielectric substrate. Basically, in a tag reading operation, the tag is mechanically displaced over the sensitive part of the reader. The reader is a planar microwave structure, typically a resonant element, coupled to a transmission line and fed by a harmonic signal. By positioning the resonator of the sensitive part of the reader at a short distance from the tag, the tag motion modulates the amplitude of the feeding signal at the output port of the transmission line (a sketch of the working principle of the proposed chipless-RFID system is depicted in Figure 11). This amplitude modulation is due to the electromagnetic coupling between the resonator of the reader and the resonators or metallic strips of the tag, which modulate the magnitude of the transmission coefficient at the frequency of the feeding signal. With respect to encoding, it can be done either by cutting those elements of the tag associated to the logic state ‘0’ or by its presence/absence at predefined equidistant positions. The former strategy is important in order to reduce costs, since it is possible to fabricate thousands of identical tags with a single mask and programming such tags in a later stage [46]. In [48], such a strategy was demonstrated since the authors implemented an 80-bit tag by means of inkjet printing on a conventional paper (see Figure 12) with all bits set to ‘1’ logic state, and then programmed with different codes by cutting those resonators associated to the logic state ‘0’. Such an approach provides, in terms of performance, high capacity and relatively small size at the expense of near-field tag reading. Nevertheless, the required proximity between the reader and the tag is not an issue in certain applications, such as secure paper (of interest to avoid counterfeiting or copying, e.g., of official documents, etc.).

**Figure 13:**
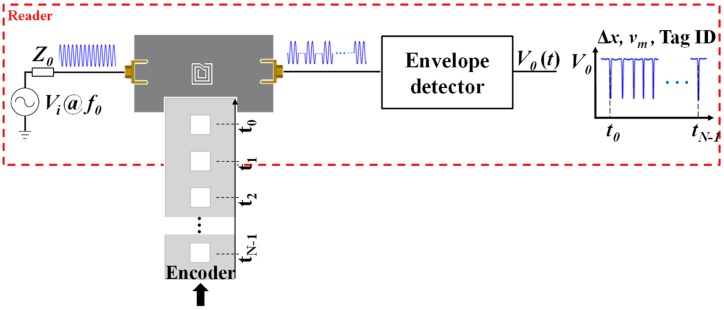
Sketch of the working principle of the all-dielectric electromagnetic encoders based on permittivity contrast. Figure extracted from [53].

**Figure 14:**
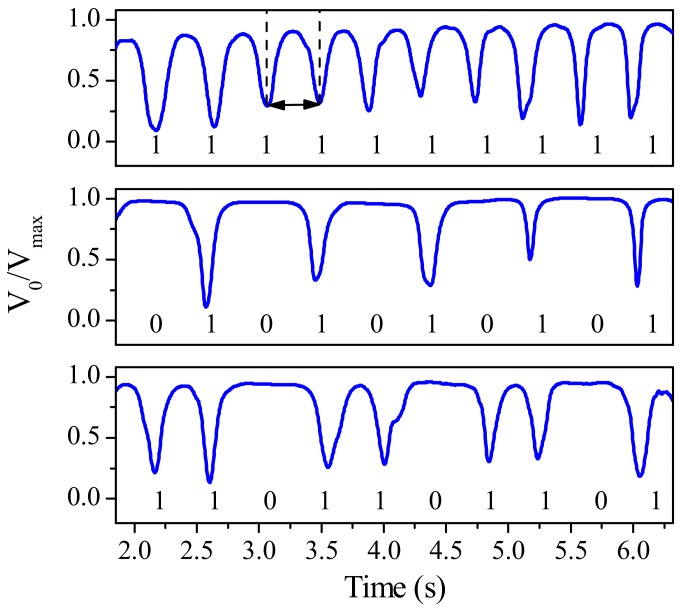
Normalized measured envelope functions of the indicated codes. Figure extracted from [53].

Recently, an alternative approach based on all-dielectric electromagnetic encoders was proposed, avoiding the use of metallic elements (either resonant element or strips). In this case, the AM signal is generated by means of permittivity contrast. Specifically, in [53], the functionality of the system was demonstrated by implementing a chain of 10 apertures on the dielectric substrate *RO4003C* with permittivity and loss tangent of 3.55 and 0.0021, respectively. The sketch of the working principle is depicted in Figure 13, whereas the normalized measured envelope functions of three different codes are shown in Figure 14.

## 3. Chipless-RFID Tags Based on Frequency Domain

Chipless-RFID tags based on frequency domain, also known as spectral signature barcodes, are implemented with resonant elements tuned at different, and predefined, frequencies. Such frequencies are distributed within a certain frequency band, which must be covered by the interrogation signal. Typically, each resonant element provides a bit of information, and the encoding is determined by the absence or presence of singularities in the amplitude and/or phase of the frequency response of the tag. According to the type of interaction between the interrogation signal and the tag, frequency domain-based tags can be classified as retransmission-based [54,55,56,57,58,59,60,61,62,63,64] and backscattered-based [65,66,67,68,69,70,71,72,73,74,75,76,77,78,79] tags.

### 3.1. Retransmission-Based Tags

**Figure 15:**
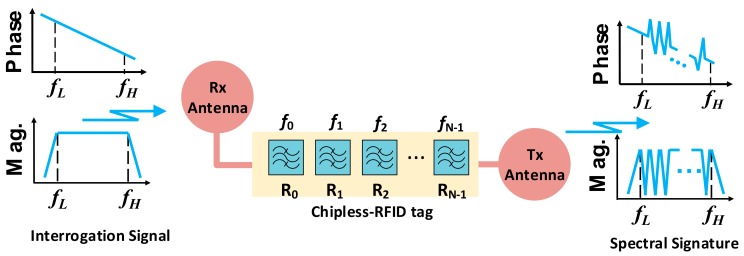
The working principle of retransmission frequency domain chipless-RFID systems.

**Figure 16:**
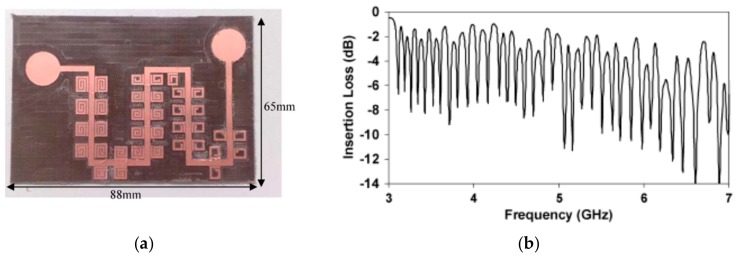
(**a**) Photograph and (**b**) measured response of a 35-bit tag implemented by Preradovic et al. Figure extracted from [56].

The working principle of retransmission-based tags is depicted in Figure 15. Such tags were designed, implemented, and patented by Preradovic et al. [55] for the first time. Typically, these chipless-RFID tags are equipped with a transmitting and receiving antenna, which are cross- polarized in order to communicate wirelessly with the reader. Such antennas are used for the reception of the interrogation signal and the transmission of the spectral signature of the tag. A tag to be highlighted is the one implemented by Preradovic et al. As shown in Figure 16a, the tag was implemented by a meander microstrip transmission line (to reduce the size) loaded with multiple resonators, specifically 35 spiral resonators, hence encoding 35 bits in a bandwidth and a surface of 4 GHz and 88 × 65 mm^2^ (including antennas), respectively. With regard to the frequency response of the tag (see Figure 16b), it presents 35 transmission zeros in the magnitude of the spectral signature, introduced by each of the elements which are part of the tag.

### 3.2. Backscattered-Based Tags

**Figure 17:**
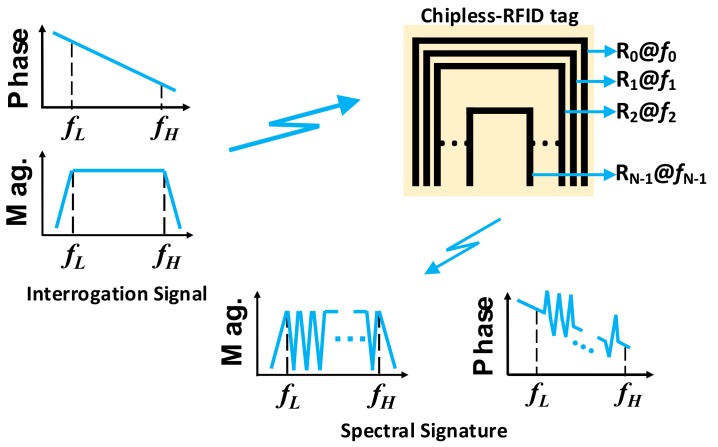
The working principle of backscattered frequency domain chipless-RFID systems.

In backscattered chipless-RFID based tags, the resonant elements provide the spectral signature through singularities in the response of the radar cross section (RCS). The main advantage of this strategy is the fact that the use of antennas is not necessary, hence the size of the tag is reduced. The working principle is illustrated in Figure 17.

In 2005, Jalaly et al. [66] proposed a very simple solution consisting of multiple dipoles with a variable capacity implemented on a substrate. The main purpose of this strategy is to create multiple resonances in the bandwidth of the tag. Similar strategy, implemented by the same authors, consists of using dipoles with variable lengths [65], making the structure completely planar. The tags are implemented in the frequency range from 2.4 to 5.8 GHz and the information capacity is 5 bits (the size of the tag is not mentioned by the authors).

**Figure 18:**
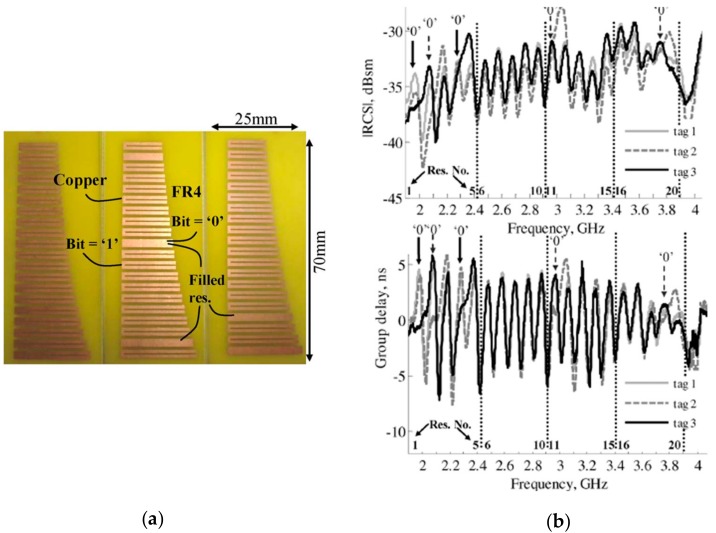
(**a**) Photograph and (**b**) measured radar cross section (RCS), and group delay of three different 20-bit tags implemented by Vena et al. Figure extracted from [69].

A tag to be highlighted is the one shown in Figure 18a and implemented by Vena et al. [69]. Such a tag is composed of 20 resonant C-shaped elements with resonance frequencies between 2 and 4 GHz, and a size of 25 × 70 mm^2^. As can be seen in Figure 18b, a peak appears in both the magnitude of the RCS and in the group delay at the resonance frequency of each resonator. With regard to the encoding, the logic state ‘0’ is achieved by shifting the resonance frequency of the resonators (C-shaped resonator) to higher frequencies. Basically, such a frequency shift is achieved by short-circuiting the resonators. The fact that it is not necessary to remove them from the tag is an important aspect, since it opens the possibility of manufacturing all the tags with all the resonators short-circuited, programming them, and removing the short-circuit with a laser, for example.

The main limitation of encoding information in the frequency domain is the bandwidth required to accommodate a large number of bits. Moreover, increasing the information capacity of the tags implies increasing the number of resonant elements, and, consequently, their size. In recent years, in order to minimize such limitations, different research groups have focused their efforts on designing tags able to encode information in more than one domain simultaneously, for example, frequency-phase, frequency-amplitude, etc. Such systems are known as hybrid systems, and the information capacity of the corresponding tags can be substantially increased, as compared to one of the frequency domain tags of this section.

## 4. Hybrid Tags

**Figure 19:**
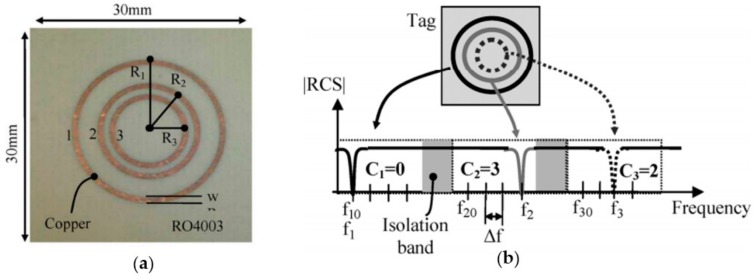
(**a**) Photograph of a chipless-RFID tag constituted by three circular rings and (**b**) the coding principle. Figure extracted from [80].

The main objective of hybrid systems is to assign more than two logic states to a single resonant element. In most cases, the approach to achieve such multi-state behavior is by encoding the information in the frequency [80,81,82,83,84]. Thus, the resonance frequency of each resonator can vary between different values within a predefined frequency window. Obviously, with this strategy, the number of states is limited by the bandwidth assigned to each resonant element. To clarify this concept, Figure 19 shows a chipless-RFID tag based on three circular rings. Such a tag operates in the frequency band from 3.1 to 10.6 GHz (i.e., in a bandwidth of 7.5 GHz which has been divided into 30 MHz slots). Therefore, there are 250 slots to be shared between three rings (80 for each one), and, consequently, 80^3^ = 512,000 different codes (19 bits) can be generated in an area of 9 cm^2^.

**Figure 20:**
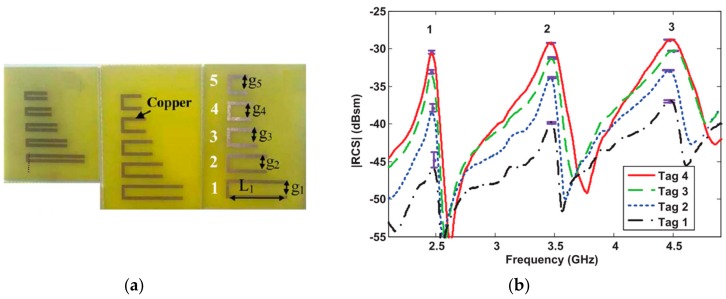
(**a**) Photograph of three chipless-RFID tags proposed by Vena et al. and (**b**) the measured frequency response. Figure extracted from [89].

Another approach to achieve a multi-state functionality is to encode the information in the amplitude level of the transmission zeros introduced by the resonant elements of the tags [85,86] or in the level of the magnitude of the resonance peaks of the RCS [87,88,89]. An example of such encoding is shown in Figure 20. The information was encoded in the magnitude level of the resonance peak introduced by the C-shaped resonator. In this case, each resonator provides 2 bits (four states) of information. The bandwidth and the area of the tag are around 3 GHz and 7.2 cm^2^, respectively.

In 2011, Vena et al. [90] presented a tag which encodes the information in two independent parameters, the phase deviation and the position of the frequency within a given frequency window.

**Figure 21:**
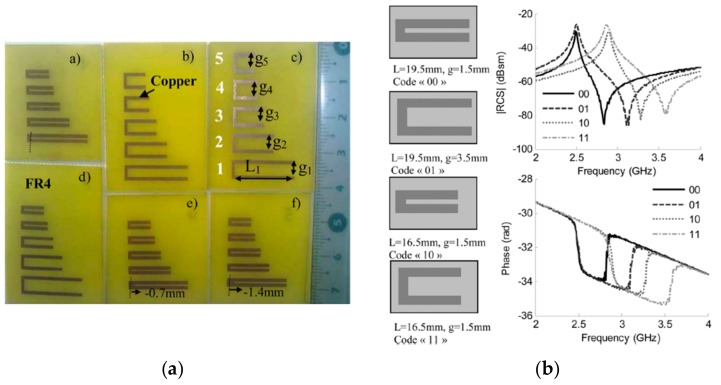
(**a**) Photograph of six chipless-RFID tags proposed by Vena et al. and (**b**) the measured frequency response. Figure extracted from [90].

Such a tag is constituted by five C-shaped resonators printed on a *FR4* substrate (Figure 21a) with resonance frequencies within the frequency band from 2.5 GHz to 7.5 GHz. Each resonator exhibits a peak and a notch controlled, basically, by the resonator length and *g*/L ratio, respectively. In the example of Figure 21b, the logic state ‘00’ is assigned to the response with a narrower phase deviation and a peak resonance at 2.5 GHz while the logical state ‘01’ corresponds to the response with a wider phase deviation and maintains the same peak resonance. The logic states ‘10’ and ‘11’ correspond to a narrow and wide phase deviation, respectively, but in this case to the peak resonance at 3 GHz. It is clear that, with this example, 2 bits of information can be assigned to each resonator. However, varying the dimensions of the resonant elements, it is possible to increase the information capacity of each particle. In fact, in the work presented by the authors, each resonator provides 24 states. Therefore, it is possible to generate 24^5^ = 7,962,654 different codes (equivalent to 22.9 bits) in a size of 8 cm^2^ with only five resonators.

**Figure 22:**
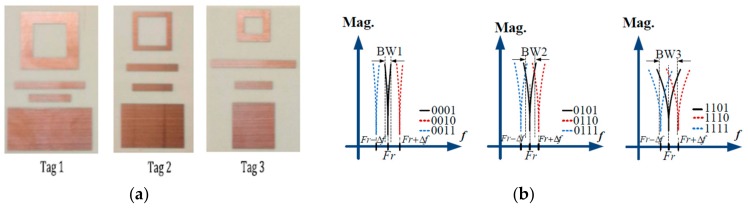
(**a**) Photograph of three chipless-RFID tags proposed by El-Awamry et al. and (**b**) the working principle of the proposed encoding. Figure extracted from [91].

Another possibility, proposed by El-Awamry et al. in [91], consists of a hybrid coding which combines the frequency position of a notch and its bandwidth. This coding technique consists of three different bandwidths (BW_1_, BW_2_, and BW_3_) and three different frequency positions (*f*_r_, *f*_r_ + Δ*f*_r_, *f*_r_ − Δ*f*), where *f*_r_ is the resonant frequency of the resonant particle and Δ*f* is the frequency offset used to encode. In order to achieve three different bandwidths, the tag uses different coding elements: a dipole, a square ring, and a patch (see Figure 22a). Consequently, the control of the position in frequency of the notch is done by varying the dimensions of the resonant elements. With this strategy, 48 bits are achieved (4 bits per resonator) in a size of 20 cm^2^ and using a bandwidth of 3 GHz.

**Figure 23:**
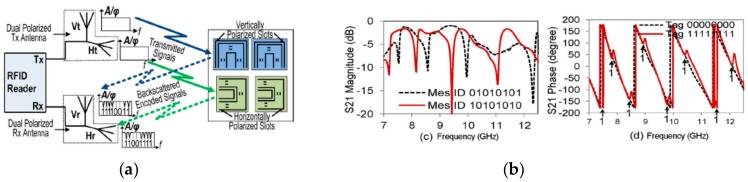
(**a**) Chipless-RFID based on polarization diversity proposed by Islam et al. and (**b**) measured frequency response of a tag with the indicated code. Figure extracted from [93].

Polarization diversity is also useful to increase the information encoded in a given surface and bandwidth [92,93,94,95]. This method is used in [93] in order to multiply by a factor of two the information capacity of the tags in the same spectral bandwidth. Such tags consist of two sets of rectangular slot resonators etched on rectangular metallic patches with different polarizations (vertical and horizontal), and in turn, each set of resonators is divided in two patches. It is important to mention that the slots etched in each patch are conveniently placed in order to reduce the coupling between resonant elements. On the other hand, in the tag reading operation, the tag must be illuminated with a plane wave orthogonally polarized (Figure 23a). Therefore, the reader consists of two dual-polarized antennas, one to transmit the interrogation signal and the other one to receive the spectral signature of the tag. With this strategy, as a proof-of-concept demonstrator, the authors encoded 16 bits within an area and bandwidth of 3.06 cm^2^ and 5 GHz, respectively.

## 5. Comparative Analysis between Coding Techniques

**Figure 24:**
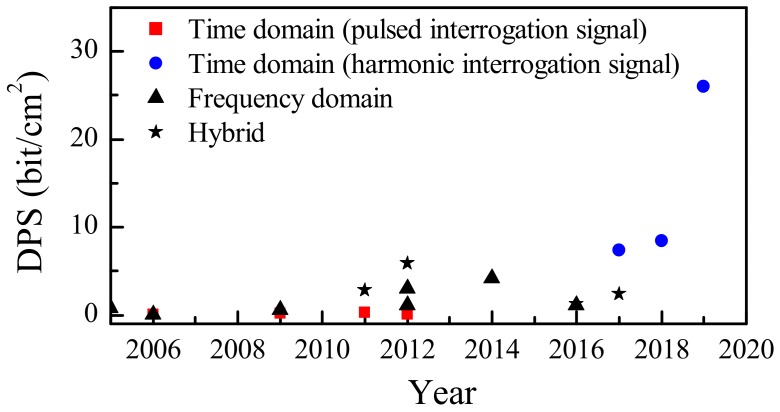
Historic evolution of DPS for different encoding methods.

As described in the previous sections, there are different parameters (tag size, bandwidth, number of bits per resonator, etc.), which must be considered in order to determine the efficiency of the coding method used. As mentioned in the introduction, the main challenges in chipless-RFID technology are: (i) to encode the maximum information in the minimum possible area, and in turn, (ii) minimizing the bandwidth. Thus, it is interesting to define figures of merit (or criteria) in order to evaluate and/or compare the different coding methods as well as tag designs. Such figures of merit measure the information density per surface (DPS) in bits/cm^2^ and density per frequency (DPF) in bits/GHz. Table 1 compares different chipless-RFID tags which can be found in the literature, including those shown and described in this paper. In general, in time domain-based tags where the interrogation signal is a pulsed signal, the DPS is very low due to the fact that it is necessary to implement transmission lines with large lengths in order to produce measurable delays. Conversely, those tags based on time division multiplexing (the interrogation signal is a harmonic signal), present, to the best of our knowledge, the largest DPS. Although this strategy provides a high data density per surface and bandwidth, it is at the expense of sacrificing read range. Nevertheless, this is not an issue in applications such as secure paper (where reading by proximity may be even convenient in order to provide major levels of confidence against eavesdropping or spying). On the other hand, typically, either frequency domain-based tags or hybrid systems need a large number of resonators in order to accommodate a significant number of bits. In fact, both encoding methods provide, in general, tags with similar DPS (either in terms of cm^2^ or *λ_g_*^2^) as can be appreciated either in Figure 24, where the historic evolution of DPS for different encoding methods is shown, or in the Table 1. However, hybrid tags provide larger DPF as compared to frequency domain tags. With regard to the read range (RR), it is difficult to compare both encoding systems (frequency domain and hybrid tags) due to the fact that, in most cases, relevant information, such as transmitted power or antenna gain, among others, are not given in the literature. Indeed, from the results of Table 1, it is difficult to identify a superior encoding system in terms of read range.

## Figures and Tables

**Table 1:** Comparative analysis between encoding techniques.

Ref./Year	BW (GHz)	Bits	Area (cm^2^)	DPF (bit/GHz)	DPS (bit/cm^2^)	DPS (bits/*λ_g_*^2^)	RR (cm)
Time Domain (Pulsed Interrogation Signal)							
[21]/2006	---	4	---	---	---	---	---
[22]/2008	---	8	---	---	---	---	---
[24]/2006	---	4	59.4	---	0.07	---	---
[26]/2012	0.05 **	2	---	40	---	---	---
[32]/2011	---	2	8.75	---	0.23	---	---
[36]/2012	---	2	70.0	---	0.03	---	---
[38]/2009	0.8 **	5	26	6.25	0.19	---	---
Time Domain (Harmonic Interrogation Signal)							
[48]/2018	*	80	9.44	*	8.47	153.3	0.025
[42]/2017	*	40	5.40	*	7.40	117.3	0.05
[52]/2019	*	100	3.84	*	26.04	412.6	0.05
Frequency Domain							
[56]/2009	3.1–10.6	35	57.2	8.97	0.61	4.790	5
[65]/2005	5–6	5	6.48	11.1	0.77	10.43	150
[76]/2016	1.9–3.1	20	17.5	16.7	1.14	48.69	20
[69]/2012	2–4	20	17.5	10.0	1.14	24.84	50
[71]/2006	0.7–0.9	5	50.1	25.0	0.10	60.23	30
[72]/2014	3.1–10.6	24	5.76	3.20	4.17	36.33	60
[81]/2012	2–5.5	9	3.00	2.57	3.00	41.74	65
Hybrid							
[89]/2016	2–5	9	7.20	3.00	1.25	18.01	60
[86]/2017	2–3	16	6.75	16.0	2.37	96.15	***
[90]/2011	2.5–7.5	22.9	8.00	4.58	2.86	22.40	45
[93]/2012	3.2–9.6	64	10.9	10.0	5.88	52.66	5

* The spectral bandwidth of this approach is virtually null due to the fact that the interrogation signal is harmonic. ** Bandwidth of the pulsed interrogation signal. *** In this approach the reader and the tag must be in contact. --- The information is not provided by the authors. Abbreviations: BW, bandwith; DPF, density per frequency; DPS, density per surface; RR, read range.
